# Advance care planning with people with dementia: a process evaluation of an educational intervention for general practitioners

**DOI:** 10.1186/s12875-020-01265-z

**Published:** 2020-09-23

**Authors:** Bram Tilburgs, Raymond Koopmans, Henk Schers, Carolien Smits, Myrra Vernooij-Dassen, Marieke Perry, Yvonne Engels

**Affiliations:** 1 Department of IQ Healthcare, Radboudumc, Nijmegen, The Netherlands; 2 Department of primary and community care, Radboudumc, Nijmegen, The Netherlands; 3 Radboudumc Alzheimer Centre, Nijmegen, The Netherlands; 4 Joachim en Anna, Centre for specialized geriatric care, Nijmegen, The Netherlands; 5 Research Group Innovating with Older Adults, Windesheim University of Applied Sciences, Zwolle, The Netherlands; 6 Department of geriatric medicine, Radboudumc, Nijmegen, The Netherlands; 7 Department of anesthesiology, pain and palliative medicine, Radboudumc, Nijmegen, The Netherlands

**Keywords:** Process evaluation, Primary care, Advance care planning, General practitioner, Dementia, Training

## Abstract

**Background:**

General practitioners (GPs) are advised to offer advance care planning (ACP) to people with dementia (PWD). In a randomized controlled trial, an educational intervention for GPs aimed at initiating and optimizing ACP proved to be effective. During the intervention most GPs were accompanied by their practice nurse (PN). To provide insights into the intervention’s successful components and what could be improved, we conducted a process evaluation and explored implementation, mechanisms of impact and contextual factors.

**Methods:**

We used the Medical Research Council guidance for process evaluations. Implementation was explored identifying reach and acceptability. We performed descriptive analyses of participants’ characteristics; selection, inclusion and intervention attendance; a GP post-intervention survey on initiating ACP; a post intervention focus group with trainers of the intervention.

Mechanisms of impact were explored identifying adoption and appropriateness. We used: participants’ intervention ratings; a GP post-intervention survey on conducting ACP; ACP documentation in PWD’s medical files; post-intervention interviews with PWD/FC dyads. All data was used to identify contextual factors.

**Results:**

The intervention was implemented by a small percentage of the total Dutch GP population invited, who mostly included motivated PWD/FC dyads with relatively little burden, and PWD with limited cognitive decline. The mechanisms of impact for GPs were: interactively learning to initiate ACP with training actors with a heterogeneous group of GPs and PNs. For PWD/FCs dyads, discussing non-medical preferences was most essential regarding their SDM experience and QoL. Some dyads however found ACP stressful and not feasible. Younger female GPs more often initiated ACP. Male PWD and those with mild dementia more often had had ACP. These characteristics and the safe and intimate training setting, were important contextual facilitators.

**Conclusion:**

We recommend Interventions aimed at improving ACP initiation with PWD by GPs to include interactive components and discussion of non-medical preferences. A safe environment and a heterogeneous group of participants facilitates such interventions. However, in practice not all FC/PWD dyads will be ready to start. Therefore, it is necessary to check their willingness when ACP is offered.

## Background

Dementia is a life limiting syndrome with a worldwide rising number of people being diagnosed per year [1, 2]. Earlier research advised dementia care to be proactive, person-centred and to focus on living and dying well [3, 4]. All these aspects of care can be improved with advance care planning (ACP) [5, 6]. ACP has recently been defined as: “the process which enables individuals to define goals and preferences for future care with family and healthcare professionals and to record and review these preferences when appropriate” [7]. ACP thereby focuses on medical and non-medical care preferences, and should not be restricted to end of life care [7–9]. Particularly in dementia, because of the deteriorating cognition, it is advised to start ACP timely. As most people with dementia (PWD) live in the community, ACP initiation by general practitioners (GPs) is most appropriate [2, 10, 11]. However, this hardly takes place.

To train GPs in timely initiating ACP with PWD, we developed an interactive educational intervention, which we evaluated in a cluster randomised controlled trial (RCT) with 38 GPs. The 19 GPs in the intervention group initiated ACP significantly more often and discussed a statistically significant larger number of medical and non-medical preferences than those in the control group. No effects were found on patient-related secondary outcome measures, such as quality of life (QoL), shared decision making (SDM), and family caregivers’ (FCs) competence [12].

The educational intervention under study consisted of multiple components [13]. For such a complex intervention, a process evaluation can help healthcare professionals, researchers and policy makers to understand what contributes to the intervention’s success, and what can be improved. In addition, a process evaluation can provide insights into the intervention’s mechanisms of impact on everyday life by exploring if study participants adopted the trained skills in daily practice and if all stakeholders found these skills appropriate [14, 15].

Also, recruiting GPs, PWD and FCs for research is challenging and often has low GPs’ participation rates and high PWD/FC dropout rates [16, 17].

It is therefore essential to identify the population reached by the educational intervention and investigate if the intervention was found acceptable for both GPs and PWD/FC dyads [18–20].

For those reasons, we aimed to explore the implementation of the educational intervention by focussing on reach and acceptability. We also aimed to explore the impact of the intervention’s mechanisms on everyday life by focussing on the adoption and appropriateness of ACP in daily practice, including the experiences of GPs, PWD and their FCs. We also defined contextual factors important to both implementation and the mechanisms of impact.

## Methods

We used a mixed methods approach and followed the Medical Research Council (MRC) guidance for process evaluations [19]. We addressed the intervention’s implementation, mechanisms of impact on everyday life and relevant contextual factors. With regard to implementation, we focussed on reach and acceptability. Reach was defined as the educational intervention having reached the intended stakeholders. For GPs this meant that they participated in the training. For PWD/FC dyads this meant that they participated in at least one ACP conversation with their GP. Acceptability was defined as the educational intervention being acceptable, as experienced by the stakeholders [19–21]. With regard to the intervention’s mechanisms of impact in daily practice we focussed on adoption and appropriateness. Adoption was defined as the participants’ intention or initial decision to employ ACP in practice. Appropriateness was defined as participants perceiving ACP relevant in daily primary care practice [19–21]. As for contextual factors, we focussed on the setting of the intervention and the characteristics of the study participants. Contextual factors can influence both implementation and mechanisms of impact [19].

### Ethical consent

The study was approved by the medical ethics committee (CMO) of the region Arnhem-Nijmegen in accordance with the Medical Research Involving Human Subjects Acts and the declaration of Helsinki (NL52613.091.15). Anonymity was assured by removing all participant information that could lead to identification from this manuscript.

### The educational intervention

Between March and June 2016, we trained 19 GPs in initiating ACP with PWD during two workshops in a small theatre. We hypothesised that first discussing near future non-medical preferences (e.g. housing, daily activities) before preferences on medical scenarios (e.g. hospital admission, or resuscitation) were discussed, would facilitate PWD’s engagement in ACP [8, 9, 22]. Discussing such preferences was therefore included in the training.

We used role playing exercises with training actors, combined with other didactic and interactive, proven effective strategies [23]. A model for shared decision making (SDM) with frail elderly was introduced by a GP specialised in this topic and was used to guide the ACP conversations [24]. Barriers and facilitators (e.g. a trust-based relationship with the GP, engaging all stakeholders, GPs’ proactive attitude, starting timely and regularly reviewing ACP) were addressed by a GP specialized in elderly care (MP) [8, 9]. Participating GPs were invited to bring a practice nurse (PN), as PNs have an important role in dementia care communication in primary care [8, 9]. A full description of the intervention is published elsewhere [12].

### Process evaluation participants

The study population consisted of GPs, PNs and PWD/FCs dyads who participated in the RCT, and all trainers who provided the educational intervention. We purposefully selected dyads from different GP practices in order to include participants with characteristics that allowed answering our research questions. Besides, we aimed to include female and male PWD and female and male FCs who had had at least one ACP conversation after their GP was trained.

### Data collection on reach and acceptability

With regard to the intervention’s reach we used the following information. Regarding GPs we registered numbers of those invited to the cluster RCT and characteristics of those who decided to participate. We documented which GP included a PN as well as GPs’ and PNs’ workshop attendance. Six months after the intervention, all intervention group GPs were invited to complete an 8-item survey. The first item explored reach as it addressed barriers to include PWD and FCs in the study, using a five-point Likert-scale (totally disagree (1) – totally agree (5)). From PWD and FCs, we registered numbers of those invited to the cluster RCT and characteristics of those who decided to participate. When PWD and FCs decided not to participate they were asked for their reasons.

With regard to exploring the acceptability of the educational intervention we used the following information. GPs and PNs were asked to complete an evaluation form after each of both workshops. This evaluation form consisted of 10 items. Nine items used a five-point Likert-scale (totally disagree (1) - totally agree (5)), rating separate training elements (e.g. the use of training actors; the heterogeneity of the group, meaning that the group consisted of GPs and PNs with different levels of experience). We considered a rating positive when participants agreed or totally agreed with an item. With the last item of the evaluation form, participants were asked to rate the complete workshop (1–10). From all trainers who provided the educational intervention we gathered qualitative data. They took part in a focus group interview, 9 months after the intervention. All were asked for written informed consent. The topic list used was established during several meetings with the research team (BT, MP, YE, RK, MVD) (Supplementary file 1). A researcher (YE), trained in interviewing, chaired the focus group.

### Data collection on adoption and appropriateness

With respect to adoption we used the following information. From GPs we used documented ACP conversations in the medical files of included PWD to determine per GP if they initiated ACP discussions. Six months after the intervention, the medical files were analysed retrospectively by two researchers who were blinded to study allocation [12]. We also used item 2 of the GP survey, addressing barriers to initiate ACP with PWD.

With respect to appropriateness we used items 3 to 8 of the GP survey and qualitative data from PWD and FCs. We invited PWD/FCs dyads from the intervention condition to participate in a semi-structured interview. All participants were asked for written informed consent. A researcher (BT), trained in interviewing, conducted each interview at PWD’s homes, assisted by a research assistant. To guide the interviews, a topic list, constructed during several meetings with the research team (BT, MP, YE, RK, MVD) was used. (Supplementary file 2). Data collected are shown in Table 1.

**Table 1:** Data used to answer our research questions.

Research aim		Operationalization	Data collected	Data source
**To explore the implementation of the educational intervention**	**Reach**	The percentage and characteristics of persons who receive or are affected by the educational intervention	1. Numbers and descriptives on GPs’, PWD’s and FCs’ cluster-RCT invitation and participation	1. Electronic database
				2. Mail from GPs
			2. Selection procedure used by GPs	3. Educational intervention attendance form
			3. Numbers on educational intervention attendance of GPs and PNs	
				4. Telephone call
			4. Reasons why PWD/FC declined study participation	5. Electronic survey completed by GPs (item 1)
			5. GPs’ barriers on cluster- RCT inclusion of PWD/FCs	
	**Acceptability**	The perception among stakeholders that the intervention is agreeable	1. GPs’ and PNs’ educational intervention evaluation	1. Educational intervention evaluation form
			2. Trainers’ educational intervention experiences	
				2. Focus group interviews with trainers
**To explore the intervention’s mechanisms impact on everyday life**	**Adoption**	The intention or initial decision to try to employ the intervention	1. Descriptives on GPs’, PWD’s and FCs who did or did not had ACP	1. Electronic database
				2. Electronic survey completed by GPs (item 2)
			2. GPs’ barriers on ACP initiation with PWD/FCs	
			3. Documented ACP conversations with PWD	3. PWD’s medical files
	**Appropriateness**	The perceived fit or relevance of the intervention in a particular setting	1. Experiences of GPs with ACP in daily practice	1. Electronic survey completed by GPs (item 3–8)
			2. ACP experiences of PWD and FCs with ACP in daily practice	
				2. Interviews with PWD and FCs

*PWD* People with dementia; *FCs* Family caregivers; *GPs* General practitioners; *ACP* Advance care planning; *cluster-RCT* cluster randomized controlled trail.

### Additional data

From all participants, demographic characteristics were collected at baseline.

### Data analyses

For all quantitative data we used descriptive statistics. With regard to reach we compared the characteristics of PWD, FCs, PNs and GPs who participated in the RCT or who declined. In addition, we analysed the first item of the GP survey on barriers for the inclusion of PWD/FC dyads.

For acceptability, we analysed PNs’ and GPs’ workshop attendance and evaluation. The focus group was audio-taped, transcribed verbatim and analysed with content analysis separately by at least two researchers using Atlas. Ti version 7 software [25]. Text fragments related to our research aim were coded. After each interview, codes from both researchers were compared and merged and a codebook was created. As a next step, the codes were combined into categories and eventually themes. Disagreements during this process were discussed with other members of the research team (MP, YE) [25].

With regard to adoption we compared GPs who did or did not conduct ACP with PWD and FCs. ACP conversations documented in the PWD’s medical file and item 2 of the GP survey were used as our source of data.

For appropriateness, we analysed items 3 to 8 of the GP survey. To analyse the interviews with PWD and FCs, we used content analyses as described above [25].

## Results

### Implementation of the educational intervention

#### Reach

We invited 1313 GPs by mail of whom 36 GPs (2.7%) agreed to participate. Characteristics of GPs who declined are unknown. Before randomisation, participating GPs contacted 182 PWD/FCs dyads. Of those, 140 dyads, (78%) gave informed consent (mean age PWD 82y, 58% female; mean age FC 69y, 65% female). For those who declined (*n* = 42; 22%; mean age PWD 84y, 56% female; mean age FC 76y, 65% female) the expected burden of participation was the most frequently mentioned reason (*n* = 10).

Item 1 of the survey was completed by 16 (84%) of the 19 GPs from the intervention group. Thirteen (68%) GPs stated they did not invite all PWD who met the inclusion criteria (age ≥ 65, any stage of dementia, FC also participating in the study) to participate. (supplementary file 3) Several reasons for this were mentioned: dementia severity (*n* = 4); PWD’s/FCs’ lack of motivation to discuss ACP (*n* = 11), PWD/ FCs not being aware or accepting the dementia diagnosis (*n* = 5), PWD/FCs denying possible future problems (*n* = 4).

#### Acceptability

Of the 19 GPs in the intervention group (range age 36-63y, 8 females), 16 (84%) attended both workshops, and three (16%) attended one workshop. Reasons for non-attendance were: lack of time (*n* = 2) and illness (*n* = 1). Of the 18 practice nurses, 15 (83%) attended both workshops. Three (17%) attended only one workshop due to time constraints.

Twenty-six participants (GPs and PNs) completed the workshop’s evaluation form. All were positive about practicing ACP with training actors. All but one (96%) were positive about the workshops’ relevance and alignment with daily practice. Twenty-one (81%) were positive about the presentations on ACP and the SDM model with frail elderly. Twenty-two (85%) were positive about the location of the workshops. Fifteen (58%) were positive about the presentation given by a FC. The workshops received a mean overall rating of 8.1 (out of 10).

The focus group interview with the seven trainers (range age 40-66y, four women) took place in November 2017 at the Radboud University Medical Centre. Data analyses resulted in two themes (the workshops’ successful elements and elements which could be improved; contextual factors) and five categories (communicating goals during ACP; from theory to practice; workshop components which could be improved; the heterogeneity of the workshop participants; the workshop environment). (Table 2).

**Table 2:** Categories and codes from the focus group interviews with trainers and training actors.

Themes	Categories	Codes
**The workshop’s successful elements and elements which could be improved**	Communicating goals during ACP	ACP should start with what is currently important in life
		Talking about life values is the essence of ACP
		Trough ACP keeping a dignified life should be discussed
		Workshops focuses on communication
		Workshop is about making contact during ACP
		A personal relation is important during ACP
		The workshops focuses on talking about remaining QoL
	From theory to practice	Addressing Some theory is necessary
		Experiencing ACP is most important
		Experiencing ACP deepens the theory
		Workshops focus on practicing ACP
		A demonstration helps to understand theory
	Workshop components which could be improved	Family caregiver presentation lacked a clear focus
		Family caregiver presentation was to personal
		The family caregiver presentation did not focus on complexity of the situation
	The heterogeneity of the workshop participants	Every group is different
		Participants had different levels of experience with ACP
		Participants had different levels of experience with dementia
		Not all participants have the same learning curve
		Heterogeneous groups enrich the workshops
		Participants learn from each other
		Participants have their own communication preferences
**Contextual factors**	The workshop environment	Small groups are important
		Maximum of five participants per training actor
		Fifteen is the maximum group size
		The intimate setting facilitates learning
		The theatre contributed to the intimate setting
		the intimate setting facilitated involvement

*ACP* Advance care planning; *QoL* Quality of life.

##### Theme 1: the workshops’ successful elements and elements which could be improved

The trainers found practicing ACP with training actors and starting ACP with non-medical preferences currently important to the PWD’s QoL, the most successful workshop elements. In addition, the trainers stated that they demonstrated that for ACP it is important to establish a personal relationship with the person with dementia and FC. Such a relationship creates an atmosphere where difficult issues concerning care preferences and maintaining a dignified remaining phase of life with optimal quality can be discussed. Balancing theoretical and interactive exercises was also considered a successful element.

*“When there was more tranquillity during an ACP conversation, and we (trainers who acted as PWD) were given the time to tell things, we actually won time and were able to discuss difficult subjects...... When a GP was rushed, you (training actor) became restless or confused. When there is tranquillity and time is taken, you get a completely different conversation which is also more pleasant.”*

Trainers stated that each participant (GPs and PNs) had a different learning curve and that their experiences with PWD and ACP prior to the training differed. This heterogeneity made the workshops challenging. Nevertheless, trainers preferred such a heterogeneous group because participants then also learned from each other.

*“I (trainer) have to say... when a group is more diverse, it gets more interesting, especially when a group is not that big, diversity is nice. To me it is not that interesting whether a participant is a PN or a GP. I just see 15 people who want to learn from each other.”*

According to the trainers, the presentation given by a FC left room for improvement, as this did not fully address the complexity of caring for a person with dementia. Looking back, the trainers found that they had not discussed the aims of her presentation thoroughly enough with the FC.

##### Theme 2: contextual factors

According to the trainers, the workshop location (a small theatre) and the limited number of participants (maximum of 15 participants with four trainers), created an intimate and safe setting. As a result, trainers were able to give sufficient personal attention and feedback and participants dared to experiment when practicing ACP conversations.

### Mechanisms of impact on everyday life

#### Adoption

Review of the medical records showed that 16 (84%) of the 19 GPs in the intervention group had had at least one ACP conversation with at least one person with dementia during the 6 months after the intervention. Nine GPs (47%) had had at least one ACP conversation with more than half of the included PWD from their practice. These GPs were younger (45.1 vs. 51.7 years) and more often female (7 out of 9 vs. 3 out of 10) compared to the 10 (53%) GPs who had had ACP conversations with less than half of the PWD from their practice. (Supplementary file 3).

In the GP survey, thirteen (68%) GPs stated they had initiated ACP with all included PWD. (Supplementary file 3) Those who did not, stated that a lack of time and dementia severity were the main reasons for not having initiated ACP.

PWD who had had at least one ACP conversation during the 6 months after the intervention were more often male (71% male vs. 29% female) compared to those who had not had ACP 53% male vs. 47% female). In addition PWD who had ACP more often had very mild dementia (14%) compared to those who had not had ACP (3%). (Table 3).

**Table 3:** Characteristics of PWD and FCs who had ACP or had no ACP.

Characteristics	PWD who had ACP (***n*** = 35)	PWD who had no ACP (***n*** = 36)
Mean age PWD (sd)	81 (6.8)	82 (5.1)
Gender PWD	25 male	19 male
Mean age FC (sd)	70 (13.8)	69 (13.8)
Gender FC	19 male	21 male
Dementia rating scale		
Very mild	5	1
Mild	14	18
Moderate	9	9
Severe	7	8

*PWD* People with dementia; *FC* Family caregiver; *ACP* Advance care planning.

#### Appropriateness

Most GPs (57%) found it important to start ACP with discussing non-medical preferences of PWD. All GPs stated that continuing ACP about medical scenarios became easier after these non-medical preferences were known. Fifteen (78%) GPs wanted to start ACP early in the disease trajectory and found engaging FCs not difficult. Nine GPs (47%) found engaging PWD not difficult (Supplementary file 3).

Ten FCs (range age 70-84y, 6 females) and two PWD (age 70 and 84y, both female) were interviewed between January and June 2017. The first two interviews, with PWD, showed that PWD had trouble remembering ACP conversations and were not able to provide information concerning our research aims. We therefore decided to conduct the remaining interviews by telephone with only the FC. After eight interviews no new codes emerged and two more interviews were conducted to confirm saturation. Two themes (experiences with discussing goals, making timely shared decisions) including four categories (discussing medical and non-medical issues, additional ACP outcomes, shared decision making, proactive behavior) were derived. (Table 4).

**Table 4:** Appropriateness: Categories and codes from the interviews with family caregivers and people with dementia.

Themes	Categories	Codes
**Experiences with discussing preferences**	Discussing medical and non-medical issues	Choices within ACP depend on the present situation
		ACP focused on medical and non-medical issues
		ACP also focused on the here and now
		ACP mostly focused on health related issues
	Additional ACP outcomes	ACP stimulates to think about the future
		ACP provides peace
		ACP provides clarity
		ACP increases trust in the healthcare provider
		ACP increases contact with the healthcare provider
		ACP increases the knowledge about dementia
		ACP makes sure their wishes are known
		ACP was not confronting
		ACP had not been useful
		ACP was confronting
		ACP was stressful
**Making timely shared decisions**	Shared decision making	ACP should be decided upon together
		Healthcare professional should also listen to family caregiver
		FC could co-decide during ACP
		FC discussed ACP with person with dementia
		FC makes ACP decisions if necessary
		FC felt equal to the GP during ACP
		Engaging PWD is difficult because cognitive decline
		PWD keep aloof during ACP
		Making decisions for PWD is sometimes difficult
		SDM did not take place
		Taking responsibility for ACP decisions is difficult
		FC doubts if person with dementia can co-decide
		PWD’s insight in their situation is limited
		ACP is not feasible because of cognition
	Proactive behaviour	ACP has to be repeated twice a year
		ACP has to be repeated annually
		FC had not thought about the future
		Proactive behaviour stimulates ACP
		GP has to take the initiative
		FC does not take the initiative
		Regular contact is important for ACP
		Discuss ACP when problems arise
		Has not thought about the future
		Does not want to think about the future
		FC does not contact the GP herself for ACP

*ACP* Advance care planning; *GP* General practitioner; *FC* Family caregiver; *PWD* People with dementia; *SDM* Shared decisions making.

##### Theme 1: experiences with discussing preferences

Most FCs indicated that starting ACP with near future non-medical care preferences suited the PWD’s needs as these preferences importantly influenced their current situation and QoL.

*“I really liked the fact that not only medical issues were discussed. I always say: when discussing well-being, all aspects of the person have to be discussed”.*

Some FCs however stated that ACP had mostly focused on the PWD’s illness and medical preferences. According to them, this was a missed opportunity. Capabilities of PWD and non-medical issues should have been addressed as well.

Most FCs found ACP important as it provided tranquility, clarity, increased their knowledge about dementia, improved the contact with their GP and increased trust in healthcare professionals. FCs also stated that the GP gained more insight in their living situation. Some FCs however found discussing future preferences confronting, stressful and not useful. These FCs only wanted to discuss care when a problem actually arose.

##### Theme 2: making timely shared decisions

FCs appreciated that they, with the person with dementia, were engaged in ACP conversations. They could both participate and co-decide. FCs felt no hierarchy between them and the GP.

“*I really felt we could co-decide. She (GP) would put it on the table, so to speak and then we start talking about it......”*

Most FCs appreciated the GPs’ proactive behaviour as FCs would not have initiated ACP themselves.

Some FCs doubted if engaging PWD in ACP was possible because of their memory problems or limited insights. If PWD were unable to make decisions themselves, FCs decided for them, which they found difficult. Most FCs found an annual evaluation of ACP sufficient, while some wanted this at least twice a year. One FC stated that during ACP, the GP gave little opportunity for SDM.

## Discussion

In this process evaluation we aimed to explore the implementation of an educational intervention for GPs and PNs about ACP with PWD and their FCS, and the intervention’s mechanisms’ impact including important contextual factors.

The intervention was implemented by a small part of the invited GPs. The participating GPs mostly reached PWD/FC dyads who were motivated and experienced relatively little burden. The intervention’s most successful elements were practicing and experiencing timely ACP with training actors in a heterogeneous group, with near future non-medical preferences and improving QoL as the starting point. The highly appreciated training was acceptable to all stakeholders. The intimate and safe environment appeared to be an important contextual factor.

Most GPs adopted ACP in daily practice. With regard to appropriateness, GPs stated that an early start of ACP including non-medical preferences facilitated ACP. PWD/FCs dyads stated that ACP, including non-medical preferences, improved SDM and was important to PWD’s current QoL. Some FCs doubted the feasibility of ACP. ACP was more often applied by younger female GPs. Male PWD and persons with mild dementia more often had ACP. We therefore consider gender of professionals, PWD and FCs, and dementia severity important contextual factors.

### Interpretation of the study in comparison with other literature

Training healthcare professionals in communication skills regarding future care has been shown effective before [26–28]. Using role models, simulations and mixed interactive and didactic education in a small and safe environment, as we did, are thereby the most effective approaches [23, 29–33]. Although a Cochrane review concluded that the overall effects of training healthcare professionals are limited, [23] our study showed that education on professional behaviour in performing ACP in daily primary care practice can be substantial. Nevertheless, a maximum implementation degree was not reached [12, 23].

It is not surprising that GPs included PWD/FC dyads of whom they thought to be capable and willing to participate in ACP [7, 34]. ACP and SDM, when aimed at deciding on future medical preferences, require the ability to imagine future scenario’s, which is difficult for PWD, especially when dementia is severe. In addition, willingness and motivation depend on the right timing, perceived barriers and subjects discussed, and are therefore not fixed states assessable at one time point [8, 35, 36]. Regularly checking PWD/FCs dyads’ willingness and motivation, and customizing ACP to the needs and capabilities of those involved, leads to more dyads being engaged and prevents that ACP is experienced as stressful or not feasible [37–41]. In addition, taking in account the role of FCs, as cognitive decline progresses and FCs are deemed to decide for PWD, is thus important [40]. As shown in our results and earlier research, including non-medical preferences in ACP facilitates ACP as these are important to PWD’s current QoL and SDM [8, 9, 42]. To provide appropriate ACP in primary care, we recommend to include such non-medical aspects in future educational interventions.

Our research showed that gender of GPs and PWD are important contextual factors. Younger female GPs more often initiated ACP than their male and older colleagues. This is congruent with previous studies which showed that younger female GPs have more knowledge and more positive attitudes towards dementia care management [43]. We also showed that male PWD more often had had ACP than female PWD. This contrasts with earlier research which found that females are more active in decision making, are more inclined to discuss a wider variety of preferences for future care, feel more empowered by discussing care preferences and believe preferences will be granted when documented [44, 45]. On the other hand, as these characteristics also apply to FCs, the mostly female FCs of the male PWD in our study will have had an important role in initiating ACP in dementia.

In contrast to the secondary outcomes of the cluster RCT, this process evaluation shows that ACP, in which discussing nearby non-medical preferences has a central role, has an impact on experienced involvement in decision making and is important to QoL. This contrast can be explained by the fact that quantitative measurements of QoL do not properly reflect those aspects of daily life individuals find important and appraise for their QoL [46–49]. Also, earlier research indicated that PWD and FCs find qualitative research methods more appropriate to assess important aspects related to their QoL [50]. Given the above, we emphasize the importance of future research on PWD’s, FCs’ and GPs’ gender, GPs’ age, and relevant more personalised measurements of QoL for PWD.

### Study strengths and limitations

This study has several strengths. With the MRC guidance for the process evaluation of complex interventions we were able to provide insights into the effective working mechanisms of the multiple components of the educational intervention and the experiences of stakeholders when gained skills were applied in daily practice [19]. We used a mixed-methods approach, included the views of PWD, FC, GPs and trainers, and included researchers with a wide range of expertise. As a result, triangulation and in-depth understanding of our research findings was achieved [51].

Our study also has some limitations. Recruiting GPs for palliative care education research is known to be difficult and in this study only a small percentage decided to participate [17]. It is known that GPs mainly participate in research they personally find important or valuable for the medical profession as a whole [52, 53]. In addition, part of the GPs doubt the feasibility of ACP in daily practice and are uncertain about how to discuss end-of-life preferences [8, 9]. This might have contributed to the GPs’ low participation rate. However, interventions aimed at relatively new and complex skills are often implemented first in a small group of motivated professionals and from there on spread to the rest of the target population [54].

We were not able to retrieve characteristics of GPs who did not respond or declined to participate. Hence, we were not able to determine if these GPs differed from the participating GPs and could not further explore possible consequences for the intervention’s external validity.

As we did not confront GPs with the difference between the mentioned and documented ACP conversations, we were not able to explain why this discrepancy was found. This can be caused by incomplete medical records which do not reflect actual medical performance [55]. It can also be caused by GPs giving socially desirable answers in the survey.

As PWD were not able to remember ACP conversations, we may not have a complete view of how ACP was applied and experienced in daily practice.

## Conclusion

We recommend to include interactive and didactic elements in future educational interventions on ACP with PWD in primary care, and focus on practicing ACP with non-medical preferences aimed at remaining QoL as a starting point. A safe learning environment and heterogeneous groups will increase learning effects. GPs’ younger age and female gender, and PWD’s male gender may positively influence ACP initiation.

In daily practice, ACP can be experienced as stressful and not feasible by some PWD. GPs should therefore check PWD/FC dyads’ willingness to be engaged and only start when they are ready. Future research on interventions to increase engagement of PWD and FCs in ACP is recommended.

We also advise future research to include a broad sample of GPs, PWD and FCs and to take into account how gender of both the professional and patient, and age of healthcare professionals, influences ACP application in daily practice. To improve further initiation of ACP in dementia, we suggest a wider implementation of our educational intervention.

## Supplementary information

**Additional file 1: Supplementary file 1.** Topic list focus group interview with workshop trainers.

**Additional file 2: Supplementary file 2**. Topic list interviews with people with dementia and family care givers.

**Additional file 3: Supplementary file 3.** Characteristics of GPs who did or did not have ACP and the items of the GP survey.

## Data Availability

The datasets used and/or analysed during the current study are available from the corresponding author on reasonable request.

## Abbreviations

ACP: Advance care planning
SDM: Shared decision making
PWD: People with dementia
FC: Family caregiver
GP: General practitioners
MRC: Medical research council
QoL: Quality of life
