# Supplementary material for: Advance care planning with people with dementia: a process evaluation of an educational intervention for general practitioners
Source: BMC Fam Pract. 2020 Sep 23;21:199. doi: 10.1186/s12875-020-01265-z (PMC7513545; doi:10.1186/s12875-020-01265-z)
Supplement: Supplementary file 1 — Additional file 1: Supplementary file 1. Topic list focus group interview with workshop trainers. [file 12875_2020_1265_MOESM1_ESM.docx]

**Supplementary file 1: Topic list focus group interview with workshop trainers**

*Start with a description of the workshop components and with both workshop aims*

*Aim of workshop 1:*

At the end of workshop 1, general practitioners and practice nurses are able to complete the steps of the model of shared decision making with frail elderly with a person with dementia and his/her family caregiver.

*Aim of workshop 2:*

At the end of workshop 2, general practitioners and practice nurses are able to discuss and report medical and non-medical issues, important to the person with dementia’s and his/her family caregiver’s quality of life, during an ACP conversation.

*Questions:*

What did general practitioners and practice nurses learn during the workshops and how did you notice?

Did you see differences between practice nurses and general practitioners and what were these differences?

Were the different workshop components executed as planned?

Which workshop components contributed most to reaching the workshop’s aims?

Which workshop components did not contribute to the workshop’s aims or what should be improved?

What should be added to the workshops?

Did general practitioners and practice nurses appreciate the workshops and how could you notice?

What are the advantages or disadvantages of using multidisciplinary groups?

What was the influence of the workshop setting and the number of participants?
