# Supplementary material for: Advance care planning with people with dementia: a process evaluation of an educational intervention for general practitioners
Source: BMC Fam Pract. 2020 Sep 23;21:199. doi: 10.1186/s12875-020-01265-z (PMC7513545; doi:10.1186/s12875-020-01265-z)
Supplement: Supplementary file 2 — Additional file 2: Supplementary file 2. Topic list interviews with people with dementia and family care givers. [file 12875_2020_1265_MOESM2_ESM.docx]

**Supplementary file 2: Topic list interviews with people with dementia and family care givers**

- How did the advance care planning conversation with your general practitioner and or practice nurse go (what went well and what could be improved)?
- Were you engaged in the conversation and could you able to co-decide?
- What did the advance care planning conversation yield?
- Do you think advance care planning is a good addition to the care delivered by your general practitioner or practice nurse and why do you think so?
