# Supplementary material for: Advance care planning with people with dementia: a process evaluation of an educational intervention for general practitioners
Source: BMC Fam Pract. 2020 Sep 23;21:199. doi: 10.1186/s12875-020-01265-z (PMC7513545; doi:10.1186/s12875-020-01265-z)
Supplement: Supplementary file 3 — Additional file 3: Supplementary file 3. Characteristics of GPs who did or did not have ACP and the items of the GP survey. [file 12875_2020_1265_MOESM3_ESM.docx]

**Supplementary file 3:** **Characteristics of GPs who did or did not have ACP and the items of the GP survey**

| **ID number of GPs who had ACP with > 50% PWD** | **PWD who had ACP** | **GP gender** | **GP age in years** | **% elderly patients in GP practice** | **Items 1-8 of the GP survey**** | | | | | | | |
| --- | --- | --- | --- | --- | --- | --- | --- | --- | --- | --- | --- | --- |
|  |  |  |  |  | **1** | **2** | **3** | **4** | **5** | **6** | **7** | **8** |
| 554 | 3/3 (100%) | v | 38 | 8 | No | Yes | 4 | 3 | 2 | 5 | 4 | 5 |
| 28 | 2/2 (100%) | v | 45 | 10 | Yes | Yes | 4 | 5 | 2 | 2 | 4 | 4 |
| 233 | 2/2 (100%) | v | 36 | 16 | Yes | Yes | 5 | 4 | 2 | 4 | 5 | 4 |
| 234 | 3/3 (100%) | m | 58 | 25 | No | Yes | 4 | 4 | 2 | 4 | 4 | 4 |
| 552*# | 1/1 (100%) | m | 51 | 11 | - | - | - | - | - | - | - |  |
| 314 | 4/4 (100%) | v | 39 | 18 | No | Yes | 4 | 4 | 2 | 4 | 5 | 4 |
| 368 | 5/5 (100%) | v | 41 | 23 | No | Yes | 5 | 2 | 2 | 4 | 4 | 4 |
| 235 | 3/4 (75%) | v | 39 | 16 | Yes | Yes | 4 | 4 | 5 | 4 | 5 | 4 |
| 511 | 3/4 (75%) | v | 59 | 20 | No | Yes | 4 | 4 | 2 | 4 | 4 | 4 |
| Totals of GPs who had ACP with >50% PWD *** | 26/28 (93%) | 77% female | Mean 45 | 16 % | n/a | 100% yes | 4(4) | 4(4) | 2(2) | 4(4) | 4(4) | 4(4) |
| **ID number of GPs who had ACP with < 50% PWD** | **PWD who had ACP** | **GP gender** | **GP age in years** | **% elderly patients in GP practice** | **Items 1-8 of the GP survey**** | | | | | | | |
|  |  |  |  |  | **1** | **2** | **3** | **4** | **5** | **6** | **7** | **8** |
| 502* | 2/5 (40%) | m | 43 | 19 | No | Yes | 5 | 5 | 2 | 3 | 4 | 4 |
| 14 | 2/5 (40%) | m | 63 | 10 | No | No | 4 | 4 | 2 | 3 | 4 | 4 |
| 450 | 1/3 (33%) | m | 59 | 20 | No | No | 5 | 3 | 2 | 4 | 4 | 4 |
| 389 | 1/5 (20%) | v | 49 | 26 | No | No | 3 | 3 | 2 | 4 | 4 | 4 |
| 646 | 1/5 (20%) | v | 45 | 12 | No | Yes | 5 | 4 | 2 | 4 | 4 | 4 |
| 397 | 1/5 (20%) | m | 46 | 28 | No | Yes | 5 | 2 | 2 | 4 | 5 | 5 |
| 394 | 1/5 (20%) | v | 52 | 24 | No | Yes | 5 | 2 | 2 | 3 | 4 | 5 |
| 459* | 0/2 (0%) | m | 43 | 15 | No | Yes | 5 | 2 | 2 | 2 | 4 | 4 |
| 244# | 0/4 (0%) | m | 57 | 12 | - | - | - | - | - | - | - | - |
| 405# | 0/4 (0%) | m | 60 | 12 | - | - | - | - | - | - | - | - |
| Totals of GPs who had ACP with ≤50% PWD *** | 9/43 (21%) | 33% female | Mean 52 | 18 % | n/a | 62.5% Yes | 5(5) | 3(2) | 2(2) | 3.5(4) | 4(4) | 4(4) |

Supplementary table: Characteristics of GPs who did or did not have ACP and items of the GP survey

GP: general practitioner; PWD: people with dementia; ACP: advance care planning

*1 medical file missing

# GP survey missing

** 1: totally disagree; 2: not agree; 3: not disagree/agree; 4: agree; 5: totally agree

- Item 1: I have nominated all PWD who met the inclusion criteria for study participation

- Item 2: I have had one or more advance care planning conversations with all included people with dementia from my practice
- Item 3: I prefer to start ACP in an early stage of dementia
- Item 4: I find it difficult to involve PWD as much as possible in ACP
- Item 5: I find it difficult to involve FCs as much as possible in ACP
- Item 6: When discussing ACP with PWD, I start with discussing goals and preferences for future care people with dementia themselves find important
- Item 7: When goals and preferences for future care are known, deciding together on future care is more easy
- Item 8: In the future I will discuss ACP with PWD

*** GP age = mean in years; % elderly patients in practice = mean %; Item 1-8 GP survey = median (modus)
